# Regulation and therapy: the role of ferroptosis in DLBCL

**DOI:** 10.3389/fphar.2024.1458412

**Published:** 2025-01-06

**Authors:** Yifan Wang, Zhengmei He, Xinyu Dong, Yiming Yao, Qiuni Chen, Yuye Shi, Yuan Deng, Quane Zhang, Liang Yu, Chunling Wang

**Affiliations:** ^1^ Department of Hematology, The Affiliated Huaian No. 1 People’s Hospital of Nanjing Medical University, Huai’an, China; ^2^ Northern Jiangsu Institute of Clinical Medicine, Nanjing Medical University, Nanjing, China; ^3^ Department of Hematology, The Huaian Clinical College of Xuzhou Medical University, Huai’an, China

**Keywords:** ferroptosis, STAT3, Nfr2, ZEB1, IKE, α-KG, DMF, APR-246

## Abstract

Diffuse large B-cell lymphoma (DLBCL) is the most common subtype of B-cell non-Hodgkin’s lymphoma (NHL), up to 30%–40% of patients will relapse and 10%–15% of patients have primary refractory disease, so exploring new treatment options is necessary. Ferroptosis is a non-apoptotic cell death mode discovered in recent years. Its occurrence pathway plays an essential impact on the therapeutic effect of tumors. Numerous studies have shown that modulating critical factors in the ferroptosis pathway can influence the growth of tumor cells in hematological malignancies including DLBCL. This review highlights recent advances in ferroptosis-related genes (FRGs), including STAT3, Nrf2, and ZEB1, and focuses on the clinical potential of ferroptosis inducers such as IKE, α-KG, DMF, and APR-246, which are currently being explored in clinical studies for their therapeutic effects in DLBCL. Correlational studies provide a novel idea for the research and treatment of ferroptosis in DLBCL and other hematological malignancies and lay a solid foundation for future studies.

## 1 Introduction

Diffuse large B-cell lymphoma (DLBCL) is the most common subtype of B-cell non-Hodgkin’s lymphoma (NHL), accounting for 30%–40% of all newly diagnosed NHL cases worldwide (Al-Hamadani et al., 2015). According to the gene expression profile, DLBCL mainly has two biological molecular forms: germinal center B-cell-like (GCB) and activated B-cell-like (ABC) (Alizadeh et al., 2000). As of 2024, the standard treatment for DLBCL remains R-CHOP and Pola-R-CHP chemotherapy (R: rituximab, C: cyclophosphamide, H: doxorubicin, O: vincristine, P: prednisone), although this method is safe and effective, up to 30%–40% of patients will relapse and 10%–15% of patients have primary refractory disease (Tavakkoli and Barta, 2023).

The concept of ferroptosis was proposed by Dixon et al. (2012) when they observed that ferrostatin-1 (Fer-1) could specifically counteract cell death induced by RAS-selective lethal (RSL) compounds. Unlike classical cell death modes such as apoptosis, necrosis, autophagy, and pyroptosis, ferroptosis is a non-apoptotic, programmed cell death mode that is iron-dependent and mediated by multiple small molecules. Recent work has identified some FRGs that were mainly involved in iron and lipid metabolism. Abnormal iron metabolism and lipid peroxidation lead to an imbalance between the generation and degradation of reactive oxygen species (ROS), which can cause oxidative stress reactions (Pizzino et al., 2017). In normal cells, ROS is mainly produced by the electron transport chain and exists at low and fixed levels (Herb et al., 2021). Excessive ROS could cause cells to undergo ferroptosis and accompany a series of changes in cell morphology, such as outer membrane rupture, nuclear integrity, reduction of mitochondrial ridges, and membrane condensation (Cao and Dixon, 2016).

Recent studies have found that the loss of histone deubiquitinase MYSM1 can induce ferroptosis and affect the function of hematopoietic stem cell, providing a new clinical approach for treating complex blood diseases (Zhao et al., 2023). Regrading the role of ferroptosis in DLBCL mainly focused on ferroptosis-related regulatory factors. By regulating these factors, the susceptibility of tumor cells to ferroptosis would be changed accordingly.

## 2 Ferroptosis regulation and its relevance in DLBCL

**FIGURE 1:**
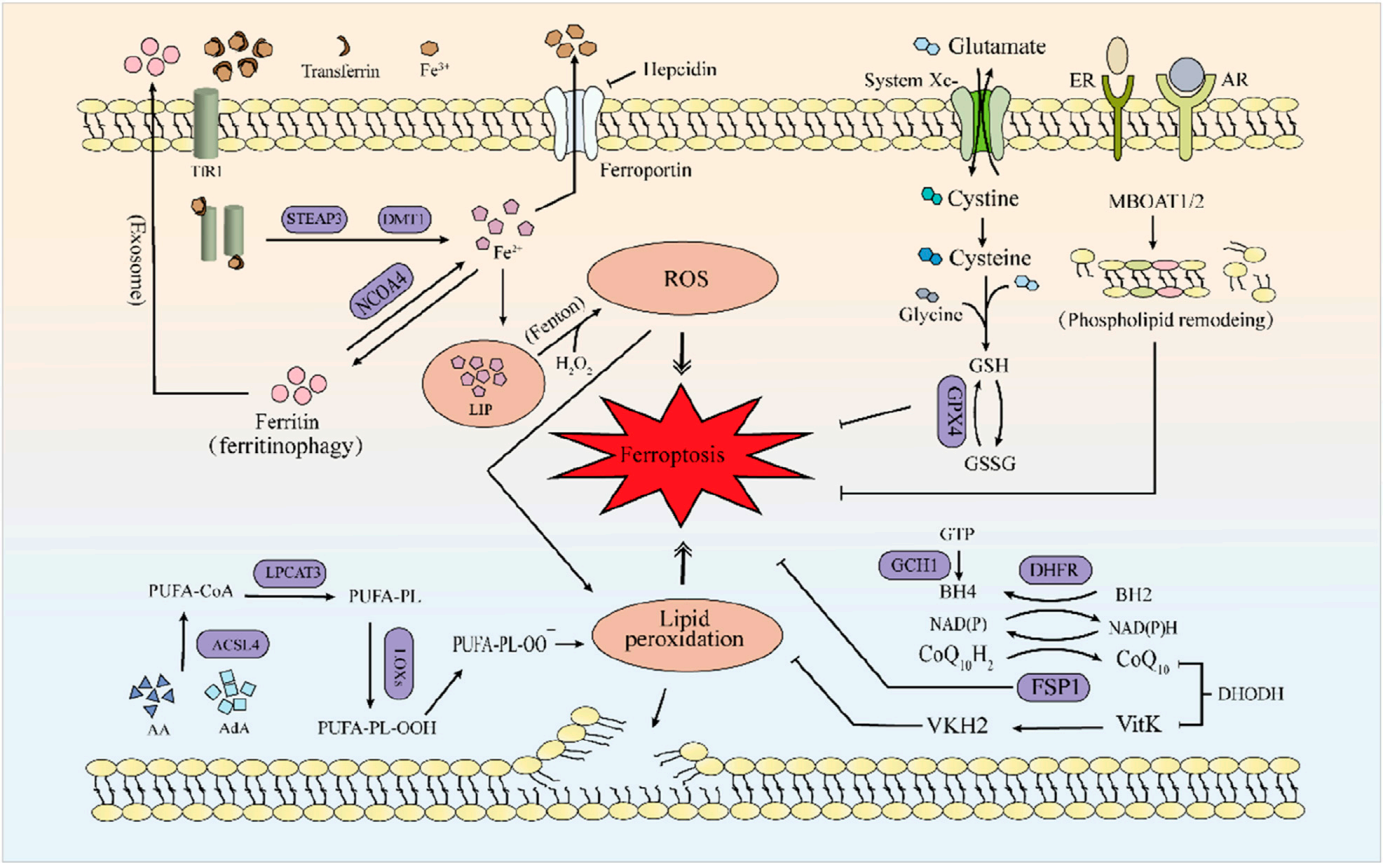
The mechanism of ferroptosis. Iron metabolism primarily involves the Fenton reaction, accumulating excessive Fe^2+^, leading to increased ROS and triggering ferroptosis. Lipids primarily composed of AA and AdA undergo lipid peroxidation catalyzed by a series of enzymes (ACSL4, LPCAT3), ultimately leading to ferroptosis. GPX4, the FSP1-CoQ10-NAD(P)H system and MBOAT1/2-mediated phospholipid remodeling can all suppress the occurrence of ferroptosis. Among these, the generation of GPX4 requires the xc-system. FSP1 can mediate the reduction of VKH2 and maintain VKH2 as an RTA to inhibit ferroptosis.

Ferroptosis is a form of regulated cell death driven by the accumulation of lipid peroxides to toxic levels, which leads to cellular damage and death. The key regulators include iron metabolism proteins and lipid metabolic enzymes like ACSL4, which promote the incorporation of polyunsaturated fatty acids (PUFAs) into membranes, making them prone to peroxidation. Unlike apoptosis or necrosis, ferroptosis is primarily characterized by iron-dependent oxidative stress. The process involves the failure of cellular antioxidant defenses, particularly the depletion of GSH and inhibition of the enzyme GPX4, which normally prevents lipid peroxidation (Figure 1) (Zhang et al., 2021a). Recent studies have highlighted the influence of epigenetic and post-translational modifications on ferroptosis regulation (Wang et al., 2023a). Non-coding RNAs (e.g., miRNAs, lncRNAs, and circRNAs) modulate ferroptosis by targeting key genes like GPX4 and SLC7A11, thereby affecting cancer cell sensitivity to chemotherapy (Wang et al., 2024). Emerging evidence suggests that the mechanisms underlying DLBCL development and progression are intimately linked to the regulation of ferroptosis. The precise processes and components underlying the mechanisms will be elaborated in the subsequent subsections.

### 2.1 Iron metabolism

Iron deficiency or iron overload can have specific adverse effects on the body (Andrews, 2000), so iron metabolism plays a vital role in the body’s homeostasis. Hepcidin, the main regulatory factor of iron homeostasis, which can induce the degradation of iron transporter protein (FPN, SLC40A1, initially also known as Ireg-1 or MTP-1), inhibiting intestinal iron absorption (Coffey and Ganz, 2017; Camaschella et al., 2020). In the case of cellular iron deficiency, divalent metal transporter 1 (DMT1, also known as DCT1 in the early stage) is upregulated (Gunshin et al., 1997). Furthermore, iron regulatory protein 2 removes iron–sulfur clusters and binds to the iron regulatory elements of target mRNA, stimulating the translation of transferrin receptor 1 (TfR1) and DMT1 and inhibiting the expression of ferritin and FPN (Anderson and Vulpe, 2009; Rouault, 2013; Hentze et al., 2010; Anderson et al., 2012).

Heme is partially degraded into Fe^2+^ under heme oxygenase-1, and Fe^2+^ enters intestinal cells after binding to heme carrier protein 1 (Choby and Skaar, 2016; Otterbein et al., 2003; Shayeghi et al., 2005). Ferritin is the principal storage form of Fe^2+^, and the surface arginine of ferritin heavy chain 1 can be re-generated into Fe^2+^ under the mediation of nuclear receptor coactivator 4 (NCOA4) (Mancias et al., 2015) a process known as ferritinophagy. Excess Fe^2+^ can form a labile iron pool (LIP) and induce ferroptosis through the Fenton reaction. FPN is the only known mammalian iron exporter to transport intracellular Fe^2+^ to the blood. Ceruloplasmin induces the conversion of Fe^2+^ to Fe^3+^ and promotes the binding of Fe^3+^ to transferrin, accelerating the mobilization of iron stores and the turnover of plasma iron (Abboud and Haile, 2000; McKie et al., 2000; Qian and Ke, 2001; Lin et al., 2024). Fe^3+^, transferrin, and TfR1 form a complex and enter the cell. The complex disintegrates under the mediation of prostate transmembrane epithelial antigen 3 (STEAP3), releasing Fe^2+^, which then enters the cytoplasm through the action of DMT1 (Fleming et al., 1997; Ohgami et al., 2005). Incorporation of iron into heme occurs within the mitochondria, where free cytoplasmic iron is utilized for the synthesis of heme and iron–sulfur clusters. Iron–sulfur clusters, as essential components of the electron transport chain complexes, facilitate electron transfer. However, electron leakage at complex I and III can lead to the generation of ROS, and excessive ROS levels may result in cellular damage or death (Read et al., 2021).

The accumulation of ROS and intracellular labile iron can enhance the sensitivity of DLBCL tumor cells to ferroptosis (Huang et al., 2023), while ROS-induced oxidative stress can activate the NF-κB pathway, which is somewhat related to the pathogenesis of DLBCL (Lingappan, 2018; Compagno et al., 2009).

### 2.2 Lipid metabolism

Arachidonic acid (AA) or its metabolite adrenic acid (AdA) is a typical free PUFA. PUFA and CoA form fatty acyl-CoA esters under the action of long-chain family member 4 of acyl-CoA synthetase (ACSL4), and fatty acyl-CoA esters form PUFA-PE with lysophosphatidylcholine acyltransferase 3 (LPCAT3) and phosphatidylethanolamine (PE) (Doll et al., 2017). PUFA-PE generates lipid hydroperoxides under the catalysis of lipoxygenase (LOX), especially 15-LOX. It is worth noting that LOX may require phosphatidylethanolamine binding protein 1 (PEBP1) to induce lipid peroxidation on the membrane (Wenzel et al., 2017). Vitamin E and tocopherol could inhibit LOX to prevent ferroptosis (Kagan et al., 2017). In addition, other oxygenases, such as NADPH oxidase (NOX) and cytochrome P450 oxidoreductase (Manea and Simionescu, 2012; Ai et al., 2021), can also cause lipid peroxidation. Free PUFA alone cannot induce ferroptosis because cells have a powerful ferroptosis defence mechanism, including glutathione peroxidase 4 (GPX4) (Gan, 2022).

Monounsaturated fatty acids (MUFA) may inhibit lipid peroxidation and ferroptosis by competing with PUFA in incorporate into phospholipids, reducing the number of PUFA-phospholipid associations. However, the lipid peroxidation reaction requires the presence of the diene-propyl portion, which exists in PUFA but not in MUFA (Magtanong et al., 2019). Therefore, MUFA itself does not induce ferroptosis. The combined action of PUFA and IFN-γ can enhance the effect of ferroptosis in tumor cells (Gan, 2022). The mechanism may be related to the increased sensitivity of tumor cells to ferroptosis by IFN-γ by inhibiting solute carrier family 7 member 11 (SLC7A11) expression (Liao et al., 2022).

Research has found that the intake of PUFAs is inversely associated with the risk of NHL, with this association being particularly prominent in DLBCL (Charbonneau et al., 2013). The lipid metabolism changes are one of the significant pathogenic factors in DLBCL (Caro et al., 2012). The latest research reveals that the immune response mechanism in DLBCL is correlated with the lipid metabolism risk model (Zhang et al., 2024).

### 2.3 The system Xc-/GSH/GPX4 axis

Organism activity is a dynamic equilibrium process, and the system Xc^−^/GSH/GPX4 axis is an important antioxidant system for ferroptosis (Li et al., 2022). Most ferroptosis inducers, such as Erastin and RSL3, act on this axis (Yang et al., 2014). The system Xc^−^ is a chloride-dependent and sodium-dependent reverse heterodimer of cysteine and glutamate located on the plasma membrane, composed of a light chain (xCT or SLC7A11) and a heavy chain 4F2hc (CD98hc or SLC3A2) (Parker et al., 2021). Among them, SLC7A11 can be regulated by critical oncogenic transcription factors, including mutant p53 (Jiang et al., 2015), Nrf2 (Sun et al., 2016), ATF4 (Chen et al., 2017), and others. The cysteine absorbed by system Xc^−^ is reduced to cysteine by thioredoxin reductase 1 (TrxR1) and then used for GSH biosynthesis (Mandal et al., 2010; Conrad and Sato, 2012). Since cysteine is the rate-limiting substrate for GSH biosynthesis and GSH is the primary antioxidant in mammalian cells, blocking the levels of cysteine and GSH in cells can directly affect the activity of GPX4 (Ursini and Maiorino, 2020). GPX4 protects cells from lipid peroxidation by converting lipid hydroperoxides (PLOOH) into the corresponding alcohols (Brigelius-Flohé and Maiorino, 2013) and plays a crucial role in regulating ferroptosis.

Lymphoid hematological diseases relatively lack glutathione peroxidase compared to normal cells, making DLBCL cells more susceptible to ferroptosis induced by system Xc-inhibitors (Iglehart et al., 1977). Since 2018, researchers have found that DLBCL cells are susceptible to ferroptosis regulated by GPX4 (Yang et al., 2014; Kinowaki et al., 2018).

### 2.4 The FSP1-CoQ10-NAD(P)H axis

Ferroptosis suppressor protein 1 (FSP1), formerly known as apoptosis-inducing factor mitochondria 2, reduces ubiquinone (CoQ10) in a way that does not depend on GSH and GPX4, captures free radicals by producing ubiquinol, induces lipid peroxidation, and prevents lipid peroxidation and related ferroptosis by directly or indirectly recycling α-tocopherol as a radical trapping antioxidant (RTA) to clear lipid radicals in the membrane. At the same time, FSP1 uses NAD(P)H to catalyze the regeneration of CoQ10 (Doll et al., 2019). As a ubiquinone reductase located on the outer surface of the mitochondrial inner membrane, dihydroorotate dehydrogenase (DHODH) is also a ubiquinone reductase. The research found that DHODH can inhibit ferroptosis by reducing CoQ10 and vitamin K (Mao et al., 2021). However, Mishima et al. (2023) proposed through experimental research that DHODH inhibitors only show significant sensitivity to ferroptosis when FSP1 is effectively inhibited, and their effect on ferroptosis is minimal. Therefore, the role of DHODH in ferroptosis is still debatable. Similar to the mechanism of action of DHODH, FSP1 can mediate the reduction of vitamin K to hydroquinone (VKH2) and maintain VKH2 as an RTA to inhibit ferroptosis and improve warfarin toxicity (Mishima et al., 2022), providing a new clinical approach for patients who cannot clot due to the use of warfarin to treat thrombosis.

### 2.5 Additional anti-ferroptosis pathways

Through CRISPR whole-genome screening, a new ferroptosis control mechanism has been identified that does not depend on GPX4 or FSP1. One is guanosine triphosphate cyclohydrolase 1, and its metabolite tetrahydrobiopterin (BH4), which mainly inhibits ferroptosis by selectively preventing phospholipid consumption, and BH4 itself is also an effective RTA (Kraft et al., 2020). Dihydrofolate reductase (DHFR) is involved in the recycling of BH4. Blocking DHFR can synergize with GPX4 inhibitors to induce ferroptosis (Soula et al., 2020). The other is sex hormone signaling, which inhibits cancer cell ferroptosis through MBOAT1/2-mediated PL remodeling (Liang et al., 2023).

## 3 Development of a ferroptosis-related gene risk scoring model for DLBCL

Up of now, the most commonly used methods for predicting the prognosis of DLBCL still include the use of the International Prognostic Index (IPI), revised IPI, and the National Comprehensive Cancer Network (NCCN-IPI). However, with the discovery that the prognosis of DLBCL patients with GPX4 positive and 8-hydroxydeoxyguanosine negative is significantly poorer in terms of OS (*p* = 0.0170) and PFS (*p* = 0.0005) (Kinowaki et al., 2018), researchers have begun to pay attention to the prognostic role of FRGs in DLBCL patients. The following study suggests that a risk-scoring model based on FRGs can be used for personalized risk assessment and prognosis prediction in DLBCL patients.

Chen et al. (2021a) used the Gene Expression Omnibus to obtain mRNA expression and matched clinical data of DLBCL patients established a personalized risk assessment model based on FRGs for DLBCL patients. However, whether these FRGs are associated with survival remains to be elusived. Subsequent studies have found that the risk-scoring model can predict the OS of DLBCL patients, with the high-risk group having lower OS. Among them, the DCA calibration curve and results have demonstrated the excellent performance of this method (Wang et al., 2023b). In addition, the model could be used for the prediction of resistance of ABC-DLBCL cells to ibrutinib treatment (Weng et al., 2022); Immune infiltration is commonly observed in the tumor cells of DLBCL (Chang et al., 2007). Concurrently, risk scores exhibit a significant correlation with immune infiltration (Xiong et al., 2022); STEAP3 plays a crucial role in the Tumor Immune Microenvironment of DLBCL (Chen et al., 2023a); the expression levels of FRGs (CAPG, HAMP, NOX4, and SLC1A5) are associated with dendritic cells, which may be significantly associated with DLBCL (Wu et al., 2023a). Previous studies have shown that dendritic cells are pivotal in anti-tumor responses (Scheuerpflug et al., 2021). In a study of tumor cells from 48 DLBCL patients, it was found that in 21% of patients, there was an increased number of CD1a+ dendritic cells at the tumor margins, and this was significantly correlated with a favorable prognosis (*p* = 0.015) (Chang et al., 2007).

Based on the relevant risk scoring models mentioned above, we have sorted out and summarized three genes that are closely related to ferroptosis. These genes are promising as new targets for DLBCL treatment and provide a strong candidate direction for future treatment strategies.

### 3.1 Signal transducer and activator of transcription 3

STAT3, as the core transcription factor in the JAK/STAT signaling pathway, was first discovered by the scientific community in 1993. This transcription factor plays a crucial role in the vital activities of cells, and can regulate key processes such as cell proliferation, survival, differentiation, migration, and immune response (Wegenka et al., 1993; O'Shea et al., 2013). The search results indicate that STAT3 plays a critical role in regulating ferroptosis in various cancer types and other diseases (Lin et al., 2024; Li and Liu, 2023; Zhang et al., 2022a).

The four splicing variants of STAT3 (α, β, △S-α, and △S-β) can all be found in DLBCL tumor cells (Turton et al., 2015). The IL-6 and IL-10 produced by ABC-DLBCL cells can lead to constitutive activation of STAT3 through autocrine action (Lam et al., 2008). This activation is an indispensable step for the survival and proliferation of ABC- DLBCL cells (Ding et al., 2008). In ABC-DLBCL patients treated with R-CHOP, STAT3 activation is closely related to poorer survival rates (Huang et al., 2013). Further studies have shown that the expression level of pSTAT3 can predict the OS and PFS of newly diagnosed DLBCL patients, suggesting its importance as a prognostic indicator (Ok et al., 2014). In mouse models, directly inhibiting STAT3 with short hairpin RNA can significantly suppress the growth of ABC-DLBCL, further confirming the critical role of STAT3 in the development of DLBCL (Scuto et al., 2011). In addition, ruxolitinib, while inhibiting STAT3 activity, also acts synergistically with lenalidomide to effectively inhibit the growth of ABC-DLBCL cells both *in vitro* and in xenograft mouse models, providing us with a new therapeutic approach (Lu et al., 2018). AZD9150, an antisense oligonucleotide targeting STAT3 mRNA, has been tested in a clinical trial involving relapsed or refractory DLBCL patients. The results demonstrated clinical benefits, including both complete and partial remissions (Shiah et al., 2021). Thus, targeting STAT3 for the development of ferroptosis-inducing drugs presents a promising research direction for treating DLBCL patients.

### 3.2 The nuclear factor erythroid 2-related factor 2

Nrf2 is a basic leucine zipper transcription factor that was first reported by Moi et al. (1994). The upregulation of Nrf2 expression can effectively inhibit the occurrence of ferroptosis, and this view has been confirmed by research (Fan et al., 2017). Conversely, when the expression level of Nrf2 decreases, the sensitivity of cancer cells to pro-ferroptosis drugs is significantly enhanced (Roh et al., 2017). The activities of glutamate-cysteine ligase, glutathione synthetase, and SLC7A11, closely related to glutathione synthesis and metabolism, are closely related to the expression level of Nrf2 in the cells, collectively maintaining the stability of the intracellular environment (Yang et al., 2005; Chan and Kwong, 2000; Habib et al., 2015; Ye et al., 2014).

The expression of Nrf2 in DLBCL tissues is significantly higher than that in reactive lymph node hyperplasia tissues, and its expression level is significantly correlated with IPI and Ann-Arbor clinical stage (*p* < 0.05) (Yi et al., 2018). Furthermore, studies have shown that the upregulation of Nrf2 expression may be related to the development of bortezomib resistance in mantle cell lymphoma (Chen et al., 2013). Further observation found that low expression of Nrf2 in the nucleus, high expression of Nrf2 in the cytoplasm, low expression of Nrf1 in the nucleus, and low expression of Keap1 in the cytoplasm are all closely related to the poor prognosis of high-risk DLBCL patients (Kari et al., 2019).

Recent studies have investigated the use of zotatifin, a clinical-stage protein synthesis inhibitor, to disrupt Nrf2’s protective role in DLBCL. When combined with ferroptosis inducers, such as IKE, zotatifin enhances the sensitivity of DLBCL cells to ferroptosis, demonstrating increased anti-cancer activity (Hsu et al., 2025; Manara et al., 2024). Thus, targeting Nrf2 in combination with ferroptosis inducers or other therapies holds potential as a novel treatment strategy for DLBCL.

### 3.3 Zinc finger E-box binding homeobox 1

ZEB1, a well-established gene associated with ferroptosis, plays a dual role in modulating this form of cell death. Depending on the specific regulatory mechanisms involved, ZEB1 can either promote or inhibit ferroptosis, highlighting its complex involvement in this process (Wu et al., 2023b; Wang et al., 2022). ZEB1 transcriptionally activates small nucleolar RNA host gene 14 and programmed death ligand 1, significantly promoting the immune evasion mechanism of DLBCL cells, thereby exacerbating the progression of the disease (Zhao et al., 2019). Furthermore, ZEB1 is considered one of the key genes closely associated with the poor clinical presentation and outcomes of DLBCL, and its aberrant expression often indicates a poorer prognosis (Lemma et al., 2013). These findings provide important clues for us to deeply understand the pathogenesis of DLBCL and develop new treatment strategies.

## 4 Therapeutic strategies targeting ferroptosis in DLBCL

**FIGURE 2:**
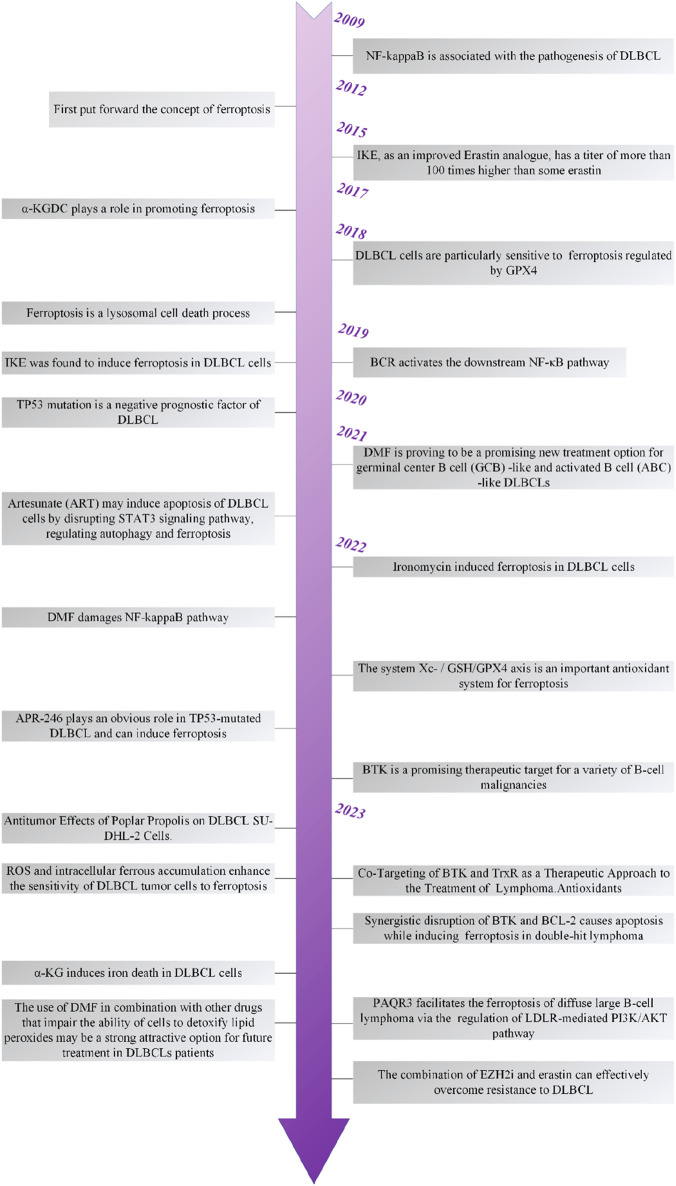
The timeline of the development of ferroptosis in DLBCL.

**FIGURE 3:**
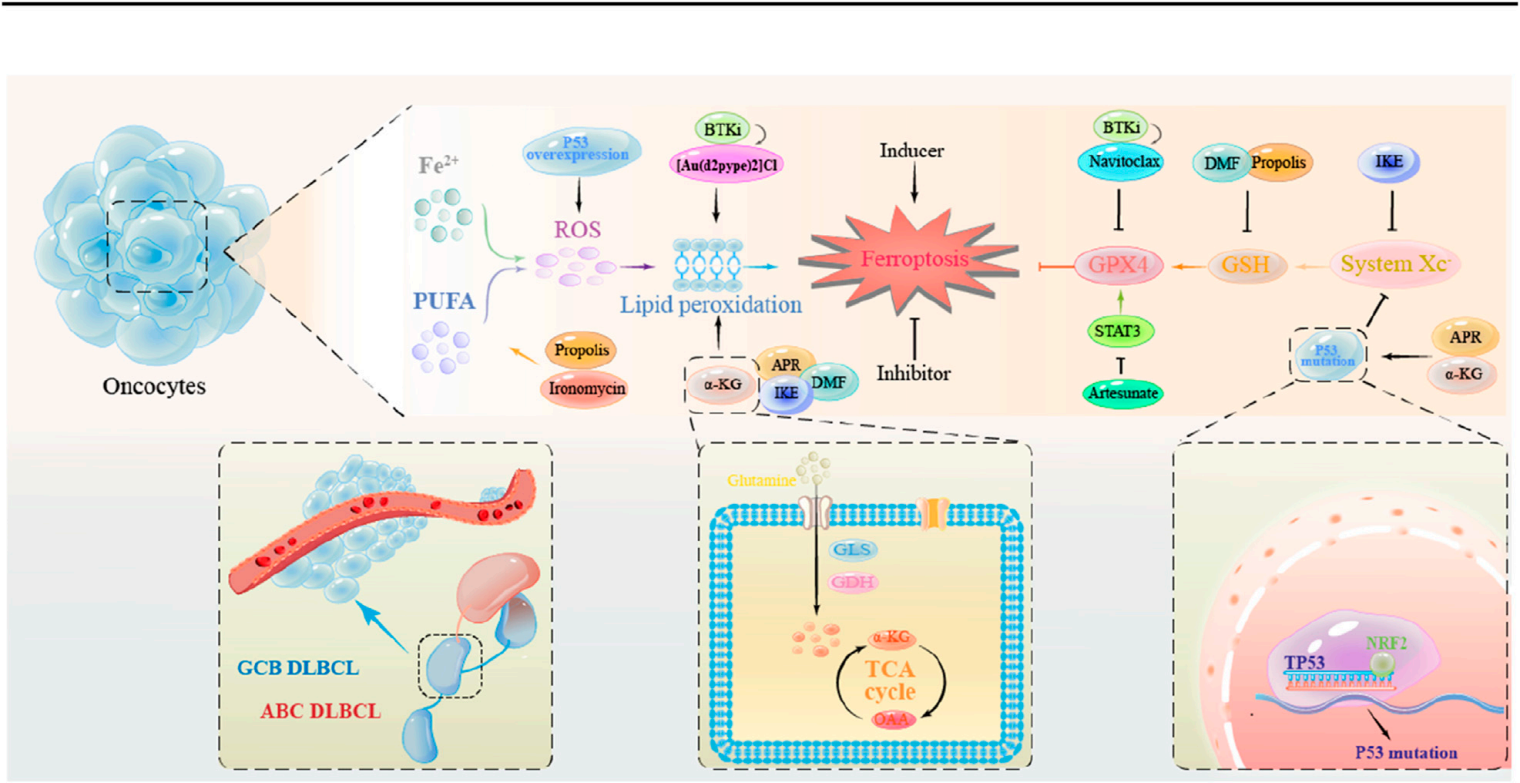
The mechanism of action of ferroptosis inducers in DLBCL tumor cells. Promotion of lipid peroxidation: APR, DMF, IKE, α-KG; Reduction of GSH synthesis: DMF; Inhibition of the System Xc-: IKE; Inhibition of the System Xc-through Mutant p53: APR, α-KG.

Ferroptosis inducers in solid and hematologic tumors were explored extensively, as shown in Table 1 (Liu et al., 2023a). Moreover, therapeutic strategies focusing on ferroptosis induction—such as inhibiting GPX4, enhancing iron metabolism, or disrupting antioxidant defenses—have shown promise in overcoming chemoresistance (Wang et al., 2023c). However, challenges like tumor heterogeneity and the complex tumor microenvironment need to be addressed. Following the understanding of the role of the ferroptosis in the pathogenesis of DLBCL (Figure 2), several ferroptosis inducers have been explored as potential ferroptosis inducers in preclinical studies for therapeutic intervention in DLBCL (Figure 3). Additionally, ferroptosis may contribute to the efficacy of R-CHOP therapy, commonly used for DLBCL. R-CHOP-induced oxidative stress and metabolic disruptions can sensitize cancer cells to ferroptosis (Hertzberg, 2022; Vitolo and Chiappella, 2023; Zhou and Busino, 2023). The development of ferroptosis-inducing therapies remains in its early stages. While preclinical findings provide valuable insights, substantial further research is required to translate these findings into clinical practice.

**TABLE 1:** Research progress and controversial points of ferroptosis-related drugs in solid and hematologic tumors.

Target	Name	Cancer	Consequence	References
The System Xc-/GSH/GPX4 axis	RSL3 FIN56 Kayadiol	Glioblastoma Colorectal cancer Glioblastoma NK/T cell lymphoma	Activation of the NF-κB pathway Direct and effective inhibitor of TXNRD1 Synergistic effect: TFEB, Torin2 The p53 pathway; A synergistic effect with L-asparaginase and cisplatin	Wu et al. (2023b) Wang et al. (2022) and Zhao et al. (2019) Lemma et al. (2013), Liu et al. (2023a), and Wang et al. (2023c) Hertzberg (2022)
	Sulfasalazine Artemisinin	Lymphoma Hepatocellular carcinoma Burkitt’s lymphoma	Not seen with sulfasalazine’s metabolites sulfapyridine and 5-aminosalicylic acid Not a inhibitor of system Xc- or other mechanisms The ATF4-CHOP-CHAC1 pathway	Vitolo and Chiappella (2023) Zhou and Busino (2023) Dixon et al. (2014)
Lipid peroxidation ROS	Statins RSL3 ART	Glioblastoma Triple-negative breast cancer Prostate cancer Ovarian cancer Pancreatic ductal adenocarcinoma	A nanoparticle system Atorvastatin improves cardiac remodeling caused by isoproterenol attack by alleviating ferroptosis The combined treatment of RSL3 and iron In cell growth arrest and killing Functional lysosomes and iron metabolism are involved in ART-induced ferroptosis in PDAC cells	Guo et al. (2018) Chen et al. (2015) Yu et al. (2015) Yu et al. (2023) Larraufie et al. (2015) Ye et al. (2020)
		Glioblastoma	Activated both the ERK and p38 signaling pathways	Zhang et al. (2019)

### 4.1 imidazole ketone erastin

Erastin, as a well-studied inducer of ferroptosis, has demonstrated efficacy in preclinical models. However, its clinical application is currently limited due to potential toxic side effects and the need for further optimization to enhance therapeutic effects. Future studies are required to address these challenges and to evaluate its safety and efficacy in clinical settings.

Erastin inhibits tumor cell growth by inhibiting the system Xc- (Dixon et al., 2014). Meanwhile, Erastin can increase the sensitivity of lung cancer cells to cisplatin (Guo et al., 2018), the sensitivity of glioblastoma cells to temozolomide (Chen et al., 2015), and the sensitivity of AML cells to anthracyclines (Yu et al., 2015). A recent experimental study found that EZH2 inhibitor can induce simultaneous upregulation of GPX4 and TfR-1, leading to resistance of DLBCL cells to EZH2 inhibitor. However, the combination of EZH2 inhibitor and erastin can effectively overcome the drug resistance of DLBCL (Yu et al., 2023).

Recently, it has been found that IKE, as an improved analogue of erastin, is more than 100 times more potent than erastin (Larraufie et al., 2015), it effectively inhibits system Xc-at low concentrations (Ye et al., 2020), making it a promising candidate for further investigation. However, its efficacy and safety profile remain to be validated in clinical trials. Zhang et al. (2019) found in a mouse DLBCL xenograft model that IKE, by inhibiting system Xc-, depleting GSH, inducing lipidomic changes through non-Fenton reaction-mediated lipid peroxidation, promotes ferroptosis, inhibits tumor cell growth. At the same time, the research used PEG-PLGA nanoparticles as carriers to combine IKE for DLBCL treatment, further increasing the therapeutic window of IKE, and concluded that the combination of IKE and other therapies may serve as a potential treatment for DLBCL.

While the preclinical data for IKE is promising, its translation into clinical practice is still in its early stages. Future research should focus on conducting rigorous clinical trials to assess its safety, efficacy, and optimal therapeutic combinations, which are crucial for determining its role in treating DLBCL.

### 4.2 APR-246

Wild-type p53 coordinates the transcription of various genes to respond to cellular stress, reduce DNA damage, and induce cell cycle arrest, senescence, or apoptosis through differential activation of target genes (Liu and Chen, 2006), preventing the replication of damaged DNA (Lim et al., 2007). Mature p53 signifies the active and stabilized state of p53 in response to cellular stressors. TP53 gene mutations impair the activity of wild-type p53, which is essentially a stress-responsive transcription factor that binds to DNA in tetrameric form (El-Deiry et al., 1992). Cells sensitive to p53-induced apoptosis produce ROS when p53 is overexpressed, cells insensitive to this mechanism cannot generate ROS (THOMAS et al., 1996). Previous studies have shown that TP53 mutations are an adverse factor for the prognosis of DLBCL, especially in GCB DLBCL (Qin et al., 2020). Accumulated mutant TP53, by binding to the main antioxidant transcription factor NRF2, inhibits the expression of SLC7A11, affects glutathione synthesis, and significantly reduces GPX4, making mutant p53 tumors more susceptible to oxidative damage (Liu et al., 2017). A recent study found that in cells with NRF2 knockout, the expression of HERC2 and VAMP8 is relatively low, ferritin and NCOA4 are increased, and low ferritin accumulates in autophagosomes, ultimately controlling iron homeostasis and increasing sensitivity to ferroptosis (Anandhan et al., 2023).

APR-246 is a small molecule that can reactivate mutant p53 in cancer cells (Lambert et al., 2009), and antagonize the thioredoxin reductase system, directly affecting the cellular redox state (Peng et al., 2013). Hong et al. (2022) found through *in vitro* and *in vivo* experiments that TP53 exon 7 mutations are associated with poorer overall survival (OS), TP53 exon 5 and 6 mutations are associated with poorer progression-free survival (PFS), and DLBCL with TP53 mutations is more susceptible to the effects of APR-246. APR-246 induces the accumulation of excess ROS, leading to ferroptosis and inhibiting tumor cell growth. Additionally, APR-246 has no significant toxicity in mice (Hong et al., 2022). The team also pointed out that in DLBCL with TP53 exon 7 missense mutations, APR-246 can induce ferritin autophagy, further triggering the Fenton reaction, increasing ROS levels, and ultimately leading to ferroptosis.

While APR-246 has shown promising preclinical results, including synergy with system Xc-antagonists in mutant p53 tumor cells, its clinical translation remains in its early stages (Liu et al., 2017). Although this compound has demonstrated efficacy in ovarian cancer models, including reducing resistance to chemotherapeutic agents like cisplatin and doxorubicin (Mohell et al., 2015), no clinical trials have yet evaluated its safety and efficacy specifically in DLBCL.

The identification of SLC7A11 as a predictive biomarker provides a rational basis for future clinical investigation into the therapeutic potential of APR-246 in DLBCL treatment. However, rigorous clinical trials are required to validate these findings, optimize dosing strategies, and assess potential toxicities before this compound can be considered a viable therapeutic option.

### 4.3 α-Ketoglutarate

In recent years, *in vitro* and *in vivo* studies have indicated that α-KG has anti-cancer effects and may serve as a potential anti-cancer drug (Abla et al., 2020). α-KG is mainly derived from glutamine and generates energy in the citric acid cycle within the cell.

Glutamine, as an essential metabolic fuel, can meet the high demand of rapidly proliferating cells for ATP, biosynthetic precursors, and reducing agents (DeBerardinis et al., 2008), with cancer cells exhibiting a greater demand for glutamine than normal cells (Yuneva et al., 2007). With the assistance of the transport system, extracellular glutamine crosses the plasma membrane and is converted to α-KG by the combined action of glutaminase and glutamate dehydrogenase. In turn, α-KG can be converted to glutamine by glutamine synthetase or to CO2 through the tricarboxylic acid cycle to provide energy for the cell (Xiao et al., 2016). Accumulated α-KG is reduced by malate dehydrogenase 1 in an acidic environment and converted to 2-hydroxyglutarate, which increases ROS levels and can induce GSDMC-dependent pyroptosis through caspase-8 activated by death receptor 6 (Zhang et al., 2021b).

Hong et al. (2022) conducted a non-targeted metabolomic analysis of serum samples from 60 newly diagnosed DLBCL patients and found that glutamine metabolism is upregulated in primary DLBCL patients and is associated with poor outcomes.

Their preclinical experiments demonstrated that α-KG induces ferroptosis by increasing ROS, promoting lipid peroxidation, and DNA oxidation damage-mediated TP53 expression, further inhibiting DLBCL tumor growth. Notably, DM-αKG treatment, a modified form of α-KG, effectively inhibited DLBCL tumor growth in mouse models, especially in cells with double hits (Cai et al., 2023). It is important to highlight that these findings are derived from preclinical models and require rigorous validation through clinical trials to confirm their translational potential.

Furthermore, it is worth noting that the α-ketoglutarate dehydrogenase complex (α-KGDC) is responsible for converting α-KG to succinyl-CoA and can generate ROS, promoting ferroptosis (Vatrinet et al., 2017), warrants additional investigation as a possible target for therapeutic intervention.

### 4.4 Dimethyl fumarate

DMF is a clinically approved drug that can impair the NF-κB/STAT3 signalling pathway in adult T-cell leukemia/lymphoma cells (MAETA et al., 2022). NF-κB and STAT3 are two transcription factors that act synergistically and play crucial roles in both inflammation and cancer (Grivennikov and Karin, 2010). STAT3 belongs to the signal transducer and activator of transcription (STAT) family of transcription factors, and it is mediated by phosphorylation of a critical tyrosine residue (Tyr 705), which induces STAT3 dimerization through phosphorylated tyrosine-SH2 domain interactions (Yang et al., 2007). Once dimerized, STAT transcription factors enter the nucleus and activate various target genes.

In preclinical models, DMF has been highlighted as a potential therapeutic agent for GCB-DLBCLs. Schmitt et al. (2021) proposed electrophiles as a new class of ferroptosis inducers, and unlike BSO and erastin, DMF induces lipid peroxidation and effectively and rapidly depletes GSH by directly inducing succinylation of cysteine residues, thereby promoting ferroptosis. Their research data suggests that the sensitivity of GCB- DLBCL cell lines to DMF-induced ferroptosis is related to their high expression of 5-LOX. Additionally, in ABC-DLBCL cells dependent on NF-κB and STAT3 survival signals, DMF can effectively inhibit the IKK complex’s and Janus kinase’s activity (Schmitt et al., 2021).

This research team found that BRD4, as a member of the BET protein family, can control the expression of FSP1 and SLC7A11, thereby protecting GCB-DLBCL cells from ferroptosis. Chromatin immunoprecipitation sequencing further confirmed this finding. The team proposed that the use of DMF in combination with other drugs that impair the cell’s detoxification of lipid peroxides may be a powerful and attractive option for the future treatment of DLBCLs (Schmitt et al., 2023). Additionally, the BRD4 inhibitor JQ1 can induce ferroptosis through ferritinophagy or by regulating the expression of iron-related genes (Sui et al., 2019). The development of combination therapies that exploit ferroptosis mechanisms represents a promising direction for future research and clinical translation.

### 4.5 Ironomycin

To some extent, Ferroptosis can be considered a form of lysosomal cell death (Gao et al., 2018). Lysosomal membrane permeabilization (LMP) is the process by which lysosomal membrane rupture leads to the release of tissue proteases and other hydrolases from the lysosomal lumen into the cytoplasm (Boya and Kroemer, 2008), and lysosomal exocytosis can reduce LMP, reducing LMP damage to normal cells (Zhong et al., 2023). Ironomycin is a derivative of salinomycin that can sequester iron in lysosomes, leading to ROS generation and ultimately causing ferroptosis (Mai et al., 2017; Versini et al., 2020).

Iron metabolism gene expression profiles in DLBCL patients has been conducted, and an iron score was established to differentiate the prognosis of DLBCL patients. Ironomycin at nanomolar concentrations can induce growth inhibition, ferroptosis, and autophagy in DLBCL cells, significantly reducing the median survival of primary DLBCL cells and has no significant toxicity to non-tumor cells (Devin et al., 2022). It is worth mentioning that significant synergistic effects were observed when ironomycin combined with doxorubicin, BH3 mimetics, and Bruton’s tyrosine kinase (BTK) inhibitors in mantle cell lymphoma (Ovejero-Merino et al., 2022). These data suggest that ironomycin is an attractive treatment strategy for DLBCL, especially in high-risk patients defined by iron score.

These findings suggest that ironomycin has potential therapeutic value in DLBCL, particularly for high-risk patients identified by the iron score. However, these observations are primarily derived from preclinical models, necessitating rigorous clinical trials to confirm ironomycin’s safety, efficacy, and combinatorial potential in DLBCL treatment.

### 4.6 BTK inhibitors

BTK is crucial in regulating the proliferation, survival, and function of B cells and plasma cells, making it a promising therapeutic target for various B-cell malignancies (Alu et al., 2022). Research suggests that BTK inhibitors may regulate ferroptosis in lymphoma and other cancers, although further studies are needed to understand the role of BTK in ferroptosis fully. BTK inhibitors have shown significant antitumor activity in various types of B cell malignancies, and their use has created new possibilities for chemotherapy-free management of these conditions.

#### 4.6.1 Ibrutinib and [Au(d2pype)2]Cl

The B cell receptor (BCR) signaling pathway can activate downstream pathways involved in cell proliferation and differentiation, including NF-κB (Pontoriero et al., 2019), ERK/MAPK (Shukla et al., 2016) and AKT (Longo et al., 2008). Bruton’s tyrosine kinase (BTK) is a crucial protein that links BCR activity to downstream pathways (Mohamed et al., 2009). By analyzing the RNA-seq dataset from TCGA, studies have indicated that the expression levels of the thioredoxin (Trx) system and the BCR signaling pathway are higher in DLBCL patient samples than in healthy samples. Knockdown of TrxR can reduce BTK mRNA and protein expression. The combination of an inhibitor of TrxR ([Au(d2pype)2]Cl) and a BTK inhibitor can activate apoptosis and ferroptosis pathways in SUDHL4 cells (Wang et al., 2023d). The combined targeted therapy of BTK and TrxR inhibitors may be an effective treatment strategy for DLBCL.

#### 4.6.2 Zanubrutinib and navitoclax

Double-hit lymphoma (DHL) is an aggressive subset of DLBCL with a poor prognosis and no satisfactory treatment options (Riedell and Smith, 2018). Zanubrutinib and navitoclax (a BCL-2 inhibitor) synergistically downregulate NRF2 and HMOX1 while inactivating GPX4, triggering ferroptosis and inhibiting the growth of DHL tumor cells. A comprehensive computational approach from single-cell to bulk analysis and *in vitro* and *in vivo* validation in DHL cell lines has demonstrated that ablating BTK enhances DHL cell sensitivity to navitoclax, inhibiting tumor cell proliferation (Setiawan et al., 2023).

### 4.7 Other potential ferroptosis inducers

#### 4.7.1 Artesunate

Artesunate (ATS) may induce apoptosis, regulate autophagy, and induce ferroptosis in DLBCL cells by disrupting the STAT3 signaling pathway (Chen et al., 202b). Based on this, the latest discovery shows that ATS has a synergistic effect with Sorafenib (Chen et al., 2023b).

#### 4.7.2 Propolis

It was found that propolis from poplar buds may inhibit the proliferation of DLBCL SU-DHL-2 cells through the ferroptosis pathway, accelerate cell death, downregulate serine/threonine-protein kinase PLK1, and affect apoptosis (Liu et al., 2023b).

## 5 Conclusion

Recent studies have shown that ferroptosis is essential in occurrence, development and the treatment of hematological malignancies (Zhou et al., 2022). Ferroptosis is critical in inhibiting tumor development and enhancing treatment efficacy (Zhang et al., 2022b). Leukemia and lymphoma are the most common hematological malignancies, and their main treatment methods include chemotherapy and stem cell transplantation. Although stem cell transplantation has improved significantly in recent years (Sakurai et al., 2023; Martin-Rufino et al., 2023), there are still certain limitations. At the same time, the remission rate of chemotherapy is low, and there has been no substantial progress in recent years. Therefore, exploring novel therapeutic approaches that are beneficial to patients remains necessary. With the continuous understanding and improvement of the mechanism of ferroptosis, inducing ferroptosis in DLBCL through different pathways is becoming a promising new option. It has been verified in animal model and *in vitro* experiments. Inducing ferroptosis in DLBCL cells, overcoming drug resistance, and combination therapy are potential applications of ferroptosis in DLBCL treatment.

Therefore, utilizing the ferroptosis mechanism and its inducers to inhibit DLBCL tumor cell growth may be a promising research direction. Ferroptosis inducers can not only enhance the efficacy of chemotherapy by inducing the death of cancer cells with low sensitivity to chemotherapy (Chen et al., 2023c) but also be used in combination with other treatments such as radiotherapy, immunotherapy, and nanotherapy to inhibit tumor cell growth further and improve efficacy. In addition to the known classic ferroptosis-related mechanisms, researchers have also found preliminary conclusions, including the use of ranolazine as an FAO inhibitor to promote ferroptosis (Sekine et al., 2022) and the regulation of low-density lipoprotein receptor-mediated PI3K/AKT pathway to promote ferroptosis in DLBCL tumor cells by progesterone and progestin receptor 3 (Song et al., 2023). However, as ferroptosis is a double-edged sword (Xu et al., 2021), researchers need to further study it to maximize its advantages and minimize its side effect. Although some clinical trials have tested the role of ferroptosis inducers in DLBCL treatment, most of the evidence is obtained from animal models, so more research is still needed to reveal its potential mechanisms and evaluate the safety and actual efficacy of human-induced ferroptosis therapy.

## Glossary

α-KG: alpha-ketoglutarate
AA: arachidonic acid
ABC: activated B-cell-like
ACSL4: long-chain family member 4 of acyl-CoA synthetase
AdA: adrenic acid
ATS: Artesunate
BCR: B cell receptor
BH4: tetrahydrobiopterin
BTK: bruton’s tyrosine kinase
CoQ10: ubiquinone
DHFR: Dihydrofolate reductase
DHL: double-hit lymphoma
DHODH: dihydroorotate dehydrogenase
DLBCL: diffuse large B-cell lymphoma
DMF: dimethyl fumarate
FPN: iron transporter protein
FRGs: ferroptosis-Related Genes
FSP1: ferroptosis suppressor protein 1
FTH1: ferritin heavy chain 1
GCB: germinal center B-cell-like
GPX4: glutathione peroxidase 4
IKE: imidazole ketone erastin
LIP: labile iron pool
LMP: lysosomal membrane permeabilization
LOX: lipoxygenase
LPCAT3: lysophosphatidylcholine acyltransferase 3
MUFA: monounsaturated fatty acids
NOX: NADPH oxidase
NCOA4: nuclear receptor coactivator 4
NHL: non-Hodgkin’s lymphoma
NKTCL: NK/T cell lymphoma
NRF2: The nuclear factor erythroid 2-related factor 2
ROS: reactive oxygen species
RSL: RAS-selective lethal
RTA: radical trapping antioxidant
OS: overall survival
PE: phosphatidylethanolamine
PFS: progression-free survival
PLOOH: lipid hydroperoxides
PUFA: polyunsaturated fatty acid
SLC7A11: solute carrier family 7 member 11
STAT: signal transducer and activator of transcription
STEAP3: prostate transmembrane epithelial antigen 3
Tf: transferrin
TfR1: transferrin receptor 1
TrxR: thioredoxin reductase
VKH2: vitamin K to hydroquinone
ZEB1: Zinc finger E-box binding homeobox 1
